# Response of *Tribolium castaneum *to dietary mannitol, with remarks on its possible nutritive effects

**DOI:** 10.1371/journal.pone.0207497

**Published:** 2018-11-14

**Authors:** Shingo Kikuta

**Affiliations:** College of Agriculture, Ibaraki University, Ami, Ibaraki, Japan; Inha University, REPUBLIC OF KOREA

## Abstract

Mannitol, one of the sugar alcohols, is often used as a low-calorific carbohydrate by animals. In some insects, mannitol acts as a cryoprotectant to endure coldness, but also become a poisonous agent. Adults of the red flour beetle *Tribolium castaneum* were shown to recognize mannitol as a factor stimulating their feeding behavior, but it remains unclear whether *T*. *castaneum* can utilize mannitol as a source of nutrition, because the enzymes needed to metabolize mannitol are unknown in this species. This study shows that *T*. *castaneum* utilizes mannitol as a nutrient in a dietary assay based on a sole carbon source added to artificial gypsum diet. The amount of mannitol excreted was less than that ingested, suggesting that it is absorbed in the insect body. The hemolymph of *T*. *castaneum* contained no mannitol but contained trehalose, a known blood sugar in insects, even after being fed mannitol. This study also revealed that dietary mannitol was metabolized to triglyceride, the main component of the fat body, forming lipid droplets. It was found that metabolites of a mannitol-supplemented diet extend the lifespan of *T*. *castaneum*, compared with those obtained by metabolizing a mannitol-free diet. Given that the insects presented transcriptional changes upon being fed carbohydrates, it might be possible to identify specific genes related to mannitol-specific metabolism by their upregulation upon mannitol intake in *T*. *castaneum*. The present study investigated mannitol-responsive gene expression using RNA-Seq. Twenty-eight genes, including those encoding trehalose-6-phosphate synthase and fatty acid synthase, were differentially expressed between beetles that were fed or not fed mannitol. The identification of upregulated genes provides us with important insights into the molecular events following mannitol intake.

## Introduction

Sugar alcohols (polyols) are used as sweeteners or sugar substitutes, providing somewhat less calories than sucrose because they are poorly absorbed in the intestinal tract [1]. Mannitol, which is the most widely distributed polyol, is commonly found in more than 50 species of plants, algae, fungi, and lichens [2, 3]. It is one of the primary photosynthetic products and increases tolerance to salt and osmotic stress in plants and algae [4–6]. These physicochemical features make mannitol suitable for use as a bulking agent during tableting in the pharmaceutical industry, because as a key component it can stabilize proteins during formulation and freeze-drying [7].

Mannitol is not widespread as a nutrient carbon source in the animal kingdom, having been reported in only a few insects [8]. Previous studies have summarized the utilization of polyols including mannitol in several insects [9, 10]. The cotton aphid *Aphis gossypii* produces and accumulates mannitol as a cryoprotectant against freezing stress [8]. In *Bombyx mori*, mannitol was less efficient as a nutrient than other saccharides [11]. In *Schistocerca gregaria* and *Locusta migratoria*, adding 11% mannitol to the diet increased their weight compared with when no mannitol was added in the diet [12], because mannitol prolonged feeding behavior. Similar results were also obtained with a coleopteran insect. The stored-product pest *Tribolium castaneum* is a serious pest in agriculture, attacking wheat flour, chocolate, and nuts, including processed foods [13, 14]. The dietary intake of *T*. *castaneum* adults was activated by some sugars and sugar alcohols. In particular, they preferred mannitol as an additive sweetener [15]. However, it remains unclear whether *T*. *castaneum* makes use of mannitol as a nutrient. Thus far, these and other studies on *Tribolium* sp. have provided descriptions related to the utilization of polyols; however, the mannitol metabolic pathway of these insects is not elucidated in detail in the general literature [9, 10]. In the Kyoto Encyclopedia of Genes and Genomes (KEGG) databases, vertebrates and invertebrates including *T*. *castaneum* are not listed as possessing genes involved in mannitol synthesis and degradation (https://www.kegg.jp. [16]). It is also possible that *T*. *castaneum* recognizes mannitol as a non-nutritive sweetener. To clarify this issue, the survival period of *T*. *castaneum* adults fed mannitol as sole carbon source needs to be evaluated.

To date, some genes for enzymes and transcription activating factors have been identified under carbohydrate-fed conditions or starvation using microarrays in *Drosophila*. Some glucosidase genes were upregulated depending on the amount of carbohydrates in the food [17]. A carbohydrate-only diet induced changes in the RNA transcript levels, which altered the storage and metabolism of fats and carbohydrates and longevity in the oriental fruit fly, *Bactrocera dorsalis* [18]. Given that insects undergo transcriptional changes upon being fed carbohydrates, some genes related to mannitol-specific metabolism are expected to be identified that are upregulated by mannitol intake in *T*. *castaneum*. Based on RNA-seq data, this study analyzed the difference in gene expressions in beetles fed diets containing mannitol and diets without carbohydrates.

## Materials & methods

### Insects

Red flour beetles, *Tribolium castaneum* (Herbst) were obtained from Sumica Technoservice Co. (Hyogo, Japan) and reared on organic whole-wheat flour (Pioneer-kikaku, Kanagawa, Japan) containing 5% yeast (Saf-instant, Lesaffre, Marcq-en-Baroeul, France) at 29 ± 1°C and 70% relative humidity under a 16L:8D cycle. Newly-emerged adults were kept without food for a week and used for dietary intake assays and RNA-seq analyses. Male and female insects were separated at the pupal stage and maintained until the adult stage for survival analyses.

### Dietary intake assay

To examine dietary intake for *T*. *castaneum* adults, the TribUTE (*Tribolium* Urges To Eat) assay was used, which measures the amount of gypsum in the excreta of beetles fed gypsum, a non-digestible and non-toxic compound [15]. Briefly, the artificial gypsum diet was comprised dry gypsum powder and water mixed in a ratio of 1.3:1 (w/w) and contained 200 mM sucrose, or mannitol solutions. The carbohydrate-free gypsum diets were not supplemented with any organic compounds, i.e. they consisted of water only. *Tribolium castaneum* adults were starved for 1week as no food was provided to facilitate their feeding behavior. A gypsum block (a cube with sides of approximately 5 mm) was provided to each beetle in a 24-well polystyrene microplate and the *T*. *castaneum* adults were kept at 25°C. The gypsum ingested by the adults was eventually excreted without digestion as a waste product that could be measured. The feces were then dissolved in 50 μL deionized water to remove carbohydrates, and the gypsum precipitate was dried thoroughly at 65°C for 24 h. The amount of gypsum eaten was measured using an analytical microbalance (AT201, Mettler-Toledo, OH, USA).

### Survival of *T*. *castaneum* adults

Each gypsum diet block of approximately 5 mm containing 200 mM sucrose, 200 mM mannitol or water only was individually provided to adults starved for 1 week and housed in individual wells on a 24-well polystyrene microplate. The set of microplates was kept at 25°C and 70% relative humidity under a 16L:8D cycle. The number of dead beetles was scored every day up to 32 days. The experiment was continued until all beetles were dead. Statistical significance was determined with a log-rank test.

### Hemolymph collection

Hemolymph was collected according to the method usually adopted for similar studies on *Drosophila* adults [19], with partial modifications for *T*. *castaneum* adults. Using tweezers, the legs of the beetles were ablated under a stereoscopic microscope on a cooling block at 0°C. Per treatment, 20 beetles were put into a 500 µL thin-walled polypropylene tube per treatment (Thermo Fisher Scientific, Carlsbad, CA, USA). The experiments were carried out in triplicate. Centrifugation was performed at 8,000 *g*, at 4°C for 20 min. The hemolymph was kept at ‒30°C until use. Male and female insects were not separated because the dietary intake was not affected by gender during the first 48 h, as described in a previous study [15].

### Carbohydrate quantification of hemolymph in *T*. *castaneum*

One microliter hemolymph from each sample was diluted with 99 µL distilled water and mixed with an equivalent amount of acetonitrile to remove the hemocytes and proteins; the solutions were centrifuged at 8,000 *g* at 4°C for 10 min before the hemolymph carbohydrate quantification analyses. The supernatant was completely dried by centrifugation at a reduced pressure. Carbohydrates were eluted with sterilized distilled water containing 1% (w/v) raffinose as an internal standard control. Carbohydrates were analyzed using HPLC equipped with a sugar separation column (shim-pack SCR101N, 7.9 mm × 300 mm; Shimadzu, Tokyo, Japan). HPLC analysis followed the previously described procedures [20].

### Quantification of triglyceride in the fat body of *T*. *castaneum*

*T*. *castaneum* adults starved for one week were fed gypsum with 200 mM mannitol or a gypsum block without carbohydrates for 48 h at 25°C. Twenty adults were dissected in sterilized PBS using tweezers, and the obtained fat body was collected into a microcentrifuge tube. The experiments were carried out in four biological replicates. Triglycerides in the fat body were quantified using a Triglyceride quantification assay kit (Abcam, Tokyo, Japan) according to the manufacturer’s instructions. The fluorescence intensity was measured using a plate reader (Infinite 200 TECAN Trading AG, Männedorf, Switzerland) installed with filters (excitation 530/20 nm, emission 600/20 nm) at 20°C. Half of the homogenized solution was used for protein measurements because the protocol of triglyceride quantification required the fat body to be resuspended in 5% NP-40 as a detergent. The protein content of the fat body was quantified using the Bradford protein assay (Bio-Rad, Hercules, CA, USA).

### Total RNA extraction and sequencing

Twenty *T*. *castaneum* adults were fed gypsum diet with 200 mM mannitol or without carbohydrates (i.e., water only) at 25°C for 48 h. Male and female insects were not separated because gender did not affect dietary intake in the first 48 h, as described in a previous study [15]. The beetles’ total RNA was isolated using ISOGEN II (NIPPON GENE, Tokyo, Japan) in accordance with the manufacturer's instructions. The quality of total RNA was confirmed using several methods and the following equipment: a Nanodrop spectrophotometer (Thermo Fisher Scientific) and a Qubit 3.0 Fluorometer (Thermo Fisher Scientific) for quantitation, agarose gel electrophoresis to observe RNA degradation and potential contamination, and an Agilent 2100 Bioanalyzer (Agilent Technologies, Santa Clara, CA, USA) to check RNA integrity and quantitation. Two Illumina-sequencing libraries were prepared using a Paired-End DNA Sample Prep kit (Illumina, San Diego, CA, USA) following standard instructions, and 150 bp paired-end reads were sequenced. Library construction, cDNA synthesis reaction, and sequencing were performed by Filgen Inc. (Aichi, Japan). The RNA-Seq library was sequenced using the HiSeq 4000 Sequencing System (Illumina). The raw sequence reads can be retrieved from the DDBJ sequence read archive (DRA) under accession number DRA007274.

### RNA-Seq analysis

Reference sequences of *T*. *castaneum* were obtained from NCBI (ID: 216). Genes were annotated using BLAST2GO Pro (BioBam Bioinformatics S.L., Valencia, Spain). Gene Ontology enrichment was analyzed using the GOSeq software [21]. TopHat v2.0.12 was used to map the reads to the *T*. *castaneum* reference genome. HTSeq v0.6.1 was used to count the read numbers mapped to single genes for quantification of the gene expression levels [22]. The expected number of Fragments Per Kilobase of transcript sequence per Million base pairs sequenced (FPKM) of each gene was calculated based on the length of the gene and read counts mapped to this gene. KOBAS v2.0 was used for gene enrichment analysis with the Kyoto Encyclopedia of Genes and Genomes databases (KEGG, https://www.kegg.jp [16]).

### Quantitative RT-PCR

A suite of processes for *T*. *castaneum* feeding assays, total RNA extraction, and cDNA transcription was performed to validate RNA-Seq. The reagents and measurement tools were identical to the ones described above. Biological samples were prepared independently of those for the RNA-Seq analysis. The single-strand cDNA was synthesized from 500 ng total RNA using PrimeScript RT reagent master mix (TaKaRa Bio, Shiga, Japan) in accordance with the manufacturer's instructions, and the concentrations and qualities were measured using a NanoPhotometer NP80 (Implen, München, Germany). Both oligo dT and Random 6 primers were used as the anchor primer. Quantitative RT-PCR was performed using SYBR *Premix Ex Taq* II (Tli RNaseH Plus, TaKaRa Bio) with a StepOnePlus (Thermo Fisher Scientific). The relative expression levels were calculated using the 2^-*ΔΔ*Ct^ method. The specificity of sequences of primers and amplified regions was confirmed by BLASTN searches against the *Tribolium* genome in the NCBI database. The internal standard gene, *RpS3* (*T*. *castaneum* ribosomal protein S3—NCBI accession: NM_001172392.1) was used as a housekeeping gene because of its expression stability through the different life stages, viz., from larva to adult [23, 24]. The primer sequences used for these analyses are shown in S1 Table.

## Results

### Effect of mannitol-supplemented gypsum diet on the longevity of *Tribolium castaneum* adults

**Fig 1:**
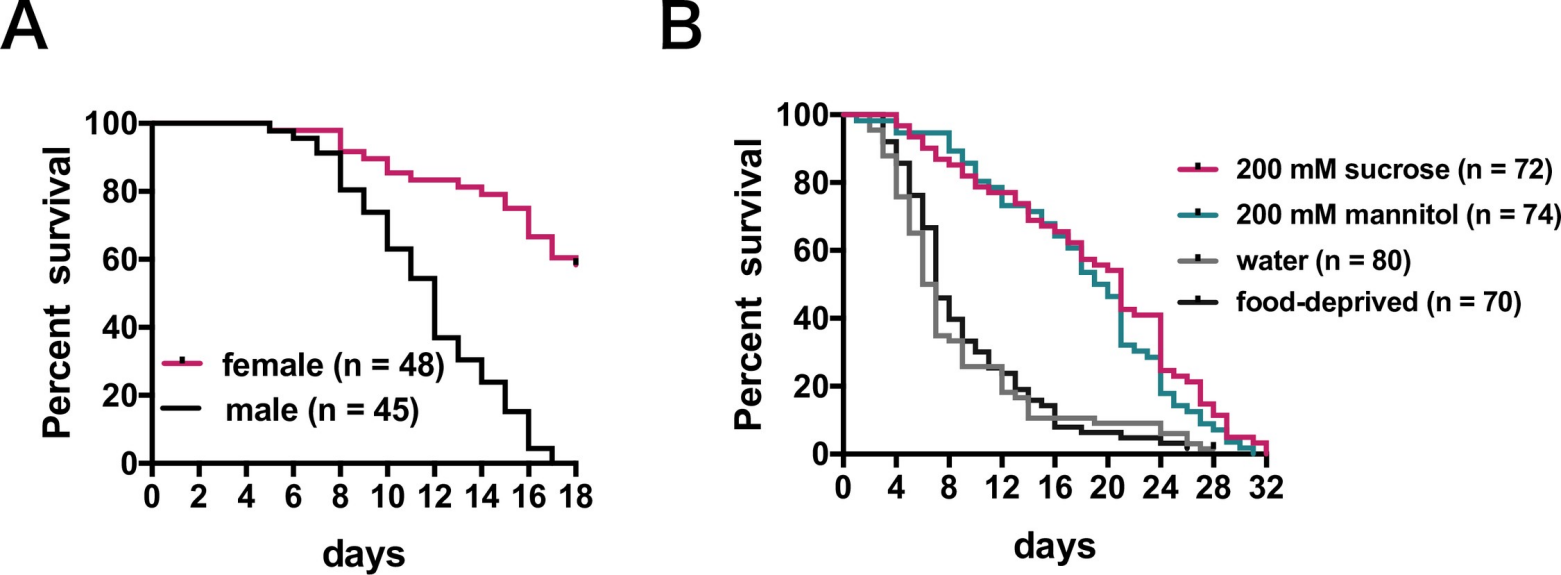
Survival of *T*. *castaneum* adults based on a gypsum diet assay. *Tribolium castaneum* adults starved for 1 week were individually fed gypsum blocks, and maintained at 25°C and 70% relative humidity under a 16L:8D cycle. (A) Comparison of male and female lifespans. The gypsum diet supplemented with 200 mM mannitol was fed to each beetle. Statistical significance was determined using the log-rank (Mantel–Cox) test (*P* ≤ 0.0001). (B) Effect of carbohydrates on the lifespan of *T*. *castaneum* adult females. 200 mM sucrose, gypsum diet in the presence of 200 mM sucrose; 200 mM mannitol, gypsum diet in the presence of 200 mM mannitol; water, gypsum without carbohydrates; food-deprived. Data were analyzed by the log-rank (Mantel–Cox) test and the Bonferroni and Sidak multiple comparison tests. Water vs. mannitol, *P* ≤ 0.0001; sucrose vs. mannitol, *P* = 0.2212 (not significant).

To assess the effect of the mannitol-supplemented gypsum diet on adult *Tribolium castaneum* longevity, by the TribUTE assay, gypsum blocks in the presence of mannitol, sucrose, or no carbohydrate were fed to *T*. *castaneum* adults. Even though the dietary intake of gypsum dosed with carbohydrates did not differ significantly between males and females over a short period of 48 h [15], longevity significantly varied between males and females when they were fed mannitol over longer 18 days (Fig 1A). Therefore, beetle survival assays were examined using females. In the absence of carbohydrates, gypsum did not affect lifespan, compared with when food was deprived, while the lifespan of *T*. *castaneum* adults was prolonged by providing gypsum dosed with 200 mM sucrose or 200 mM mannitol was provided. Longevity was not significantly different under these latter conditions (Fig 1B). Sucrose, a disaccharide composed of glucose and fructose, is metabolized as carbohydrate source. These results indicate that *T*. *castaneum* can use mannitol as a carbon source as well as sucrose.

### Consumption of mannitol

**Fig 2:**
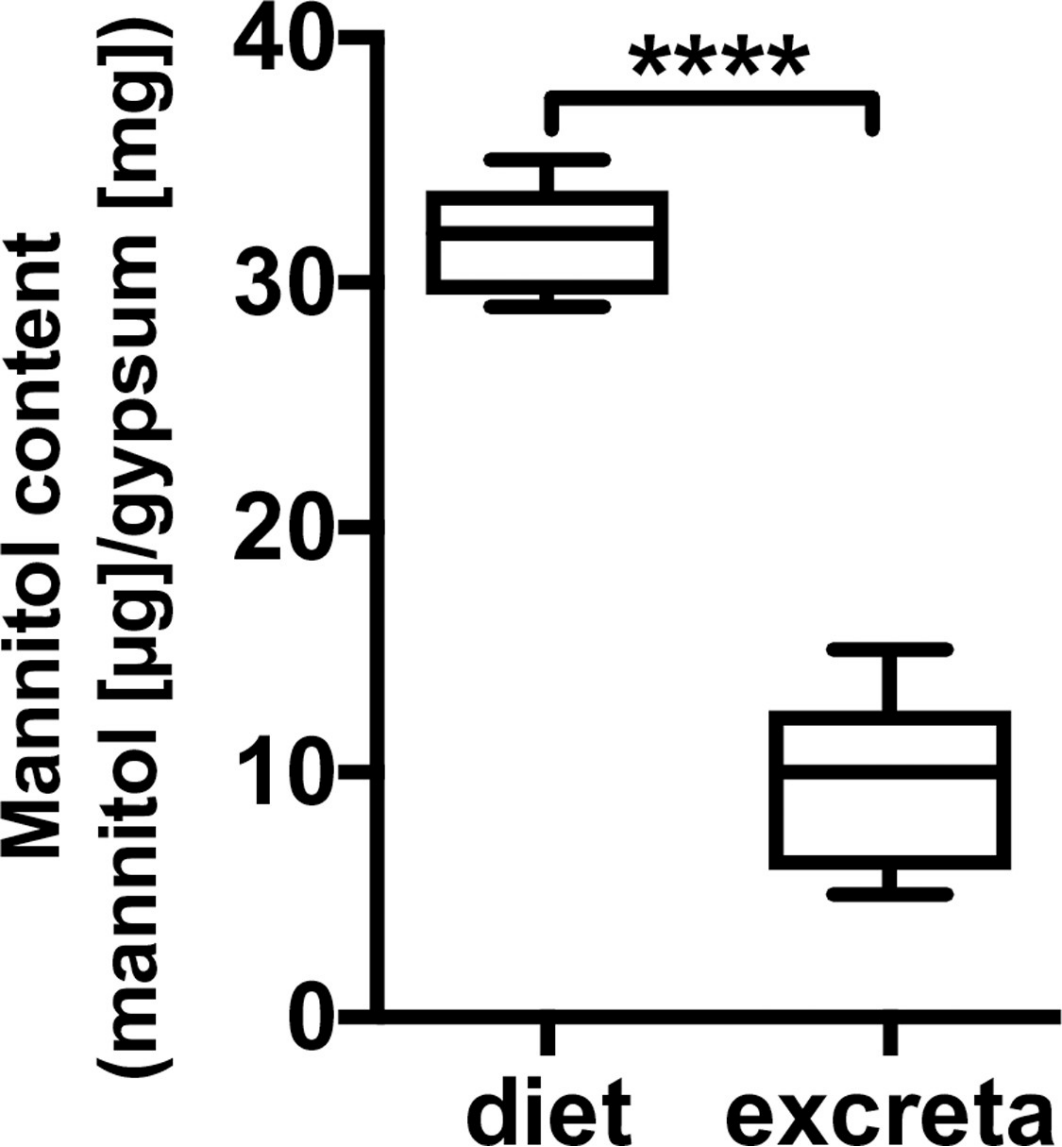
Quantification of mannitol in the gypsum diet and the excreta. The amount of mannitol in the gypsum diet and in the waste products excreted by *Tribolium castaneum* adults are shown as box plots (the highest value, upper quartile, median, lower quartile and the lowest value). *T*. *castaneum* adults were starved for 1 week to enhance the feeding behavior. *T*. *castaneum* adults were fed on the gypsum diet supplemented with 200 mM mannitol for 48 h, and the excreta of individuals (*n* = 8) were collected for mannitol quantification. Error bars show S.D. Statistical significance was determined using the *t*-test ("****" *P* ≤ 0.0001). The result presented is representative of several separate experiments. The complete set of data obtained for the biological replicates is given in the supplemental data.

The mannitol content of gypsum diet supplemented with 200 mM mannitol and of the feces of *T*. *castaneum* adults were measured. The amount of mannitol in the diet was 31.2 ± 0.8 µg/mg gypsum; in the excreta it was significantly decreased, 11.3 ± 1.0 µg/mg gypsum (Fig 2), showing that mannitol was transported across the gut to the hemolymph, or converted to other metabolites in the gut cavity.

### Carbohydrates in hemolymph

**Fig 3:**
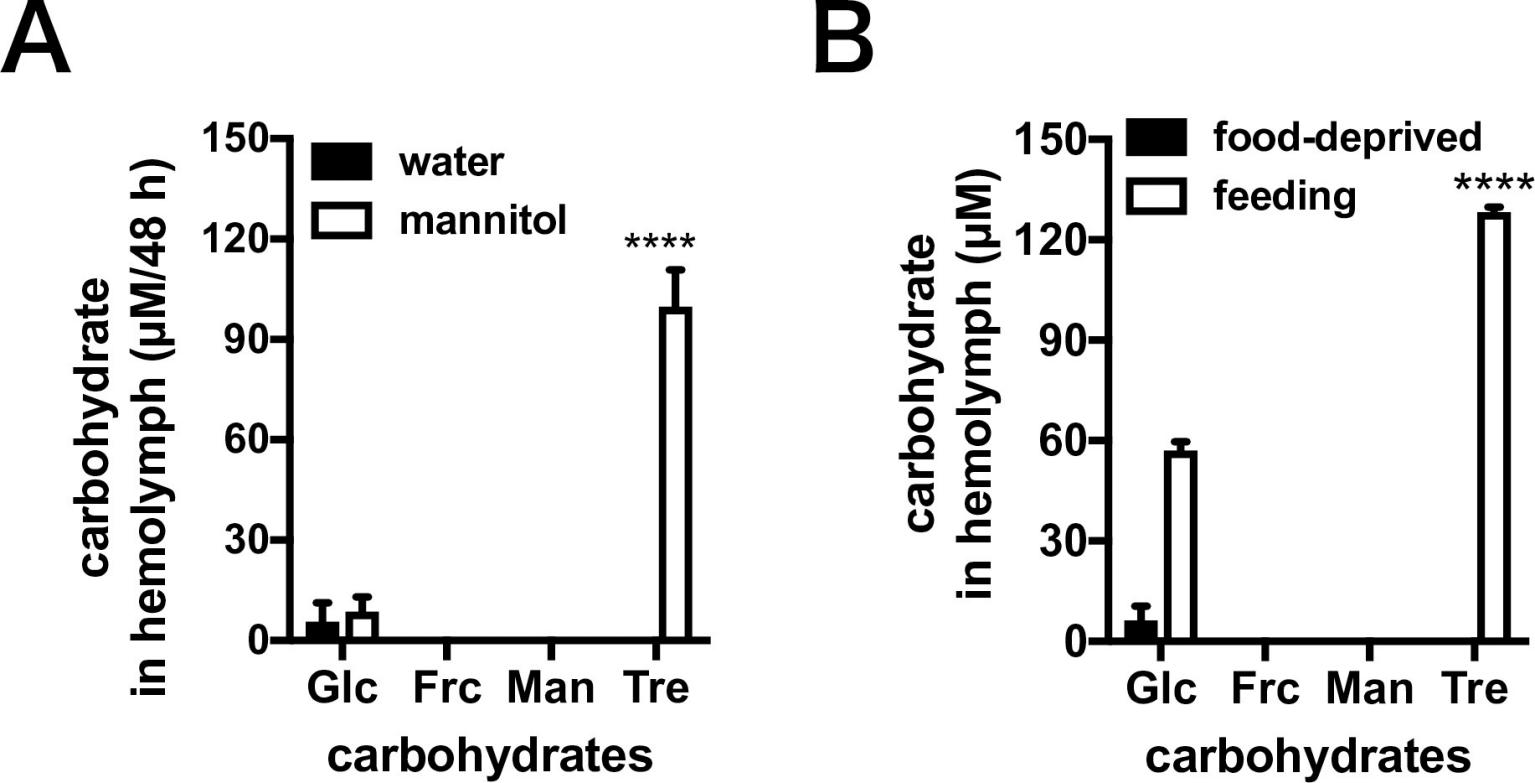
Quantification of carbohydrates in hemolymph of *T*. *castaneum* adults. *Tribolium castaneum* adults were starved for a week to enhance the feeding behavior. Glc, glucose; Frc, fructose; Man, mannitol; Tre, trehalose. Data are the mean ± S.D. The result presented are representative of several separate experiments. (A) *T*. *castaneum* adults were given gypsum diet supplemented with 200 mM mannitol or without carbohydrates (water only) for 48 h. The hemolymph was obtained following the dietary intake of gypsum. Statistical analyses were performed using two-way ANOVA [*F*(DFn, DFd): *F*(3, 16) = 56.86, *P* ≤ 0.0001] and the post-hoc Sidak's multiple comparisons test (****, *P* ≤ 0.0001) to compare the diet treatments and carbohydrates as factors. (B) Carbohydrates in the hemolymph under conventional diet. "Food-deprived" are results obtained for *T*. *castaneum* adults after starvation for a week, "feeding" means the *T*. *castaneum* adults were fed conventional food, whole-wheat flour containing 5% yeast. Statistical analyses performed were a two-way ANOVA [*F*(DFn, DFd): *F*(3, 16) = 524.9, *P* ≤ 0.0001] and the post-hoc Sidak's multiple comparisons test (****, *P* ≤ 0.0001).

Carbohydrates in the hemolymph were quantified after *T*. *castaneum* adults were fed gypsum diet supplemented with 200 mM mannitol. Mannitol disappeared from the hemolymph (Fig 3A). When *T*. *castaneum* adults were fed gypsum diet in the absence of carbohydrates (water only), trehalose, which is known as a hemolymph sugar, was not detected in the hemolymph because the trehalose would have been utilized as carbon source during starvation (Fig 3B). When *T*. *castaneum* adults were fed gypsum diet supplemented with mannitol for 48 h, trehalose was detected in the hemolymph (Fig 3A).

### Triglyceride contents in the fat body of *T*. *castaneum*

**Fig 4:**
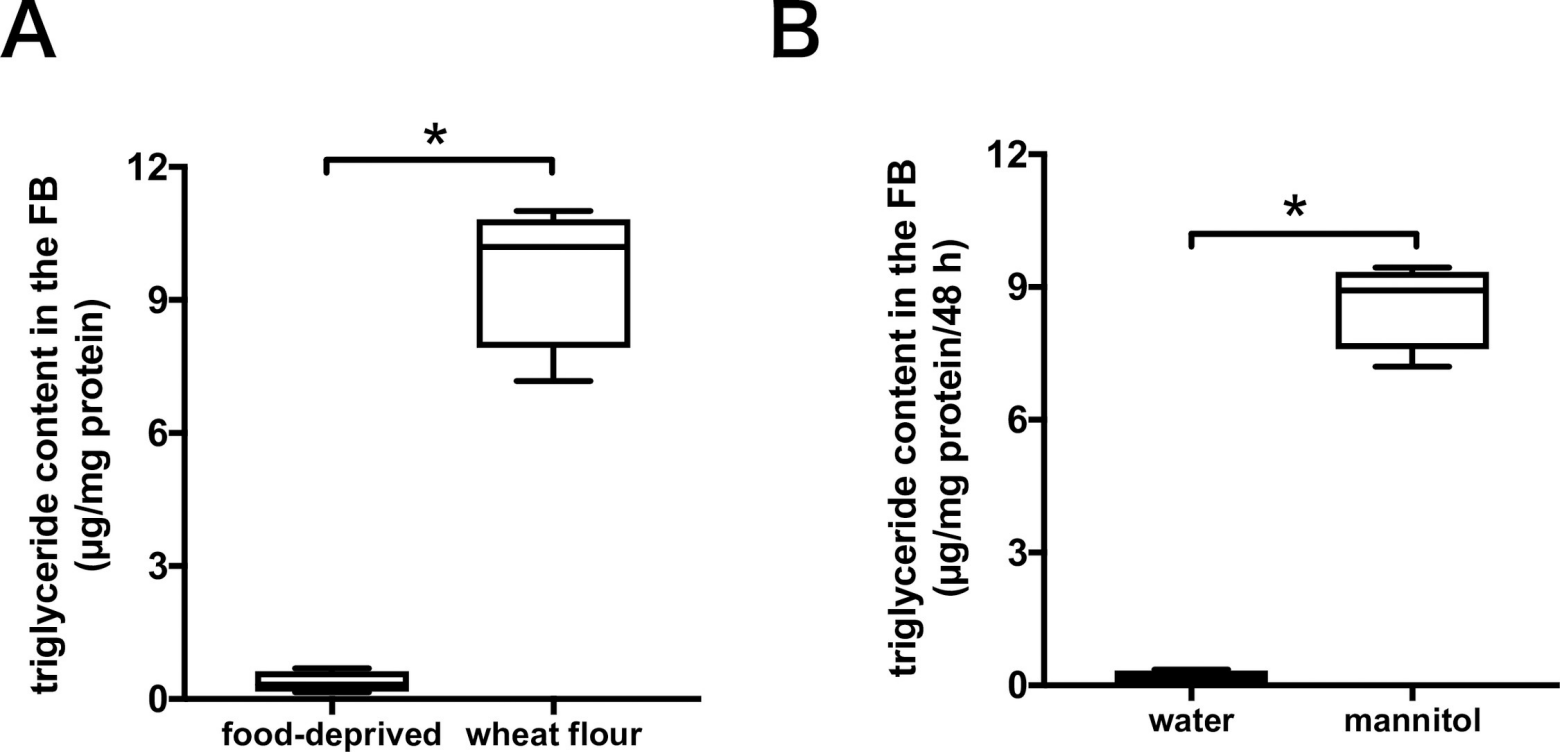
Quantification of triglyceride in the fat body of *T*. *castaneum* adults. *Tribolium castaneum* adults were starved for a week to enhance feeding behavior. Error bars show S.D. The result presented is representative of several separate experiments shown as box plot (*n* = 4; the highest value, upper quartile, median, lower quartile and the lowest value). *T*. *castaneum* adults were dissected in sterilized PBS using tweezers, and the fat bodies (FB) were collected into microcentrifuge tubes. (A) "Food-deprived" are results obtained for *T*. *castaneum* adults after starvation for a week, "feeding" means the *T*. *castaneum* adults were fed conventional food, whole-wheat flour containing 5% yeast. Statistical significance was determined using the Mann–Whitney *U* test (*, *P* = 0.0286). (B) *T*. *castaneum* adults were given gypsum supplemented with of 200 mM mannitol or without carbohydrates (water only) for 48 h. Statistical significance was determined using the Mann–Whitney *U* test (*, *P* = 0.0286).

Dietary carbohydrates are metabolized to triglyceride, which forms the main component of the fat body, in the form of lipid droplets [25–27]. The triglyceride content in the fat body of *T*. *castaneum* adults after being food-deprived for seven days was 0.4 ± 0.2 µg/mg protein. In contrast, the triglyceride content in the fat body was 9.7 ± 1.7 µg/mg protein when *T*. *castaneum* was fed wheat flour. Food deprivation resulted in the loss of triglyceride from the fat body of *T*. *castaneum* (Fig 4A). After seven days food deprivation, individual *T*. *castaneum* adults were fed gypsum dosed with 200 mM mannitol or in the absence of organic compounds for 48 h. The triglyceride content in the fat body was 0.2 ± 0.1 µg/mg protein when *T*. *castaneum* was fed gypsum without organic compounds. The triglyceride content in the fat body was 8.6 ± 1.0 µg/mg protein when *T*. *castaneum* was fed gypsum with mannitol (Fig 4B). These results indicate that feeding the beetles with gypsum supplemented with 200 mM mannitol resulted in an increased level of triglyceride content in the fat body compared to the control.

### Summary of RNA-Seq data

To examine whether gene expression depended on mannitol feeding conditions, transcriptomic data were obtained using RNA-seq. In this study, two types of cDNA sequence libraries were constructed using 20 *T*. *castaneum* adults (sex ratio: 1:1) reared carbohydrate-free (C) by giving them gypsum with water only or fed with gypsum supplemented with 200 mM mannitol (M), respectively. A total of 25,203,990 raw reads for carbohydrate-free and 25,708,420 raw reads for mannitol were generated in each sample. After removing reads containing adapters and low-quality reads from the raw data, clean reads were obtained (S2 Table). The error rate, Q20, Q30 and GC content of the clean reads were obtained, resulting in 24,744,966 (C) and 24,303,030 (M) clean reads. Using TopHat v2.0.12, 57.09% of total reads for M and 55.82% for C were mapped to the *T*. *castaneum* reference genome, respectively. In general, this number should be larger than 70% when there is no contamination and the correct reference genome is chosen. The unmapped reads were found to encode wheat genes despite the adults not feeding on these plants (DRA ID: DRA007274). The reads were removed before performing further analyses. The statistical results then indicated that the percentage of reads mapped to reference genome exons was 97.3% for mannitol (M) and 96.8% for the carbohydrate-free condition (C) (S3 Table).

### Mannitol-induced transcripts

**Fig 5:**
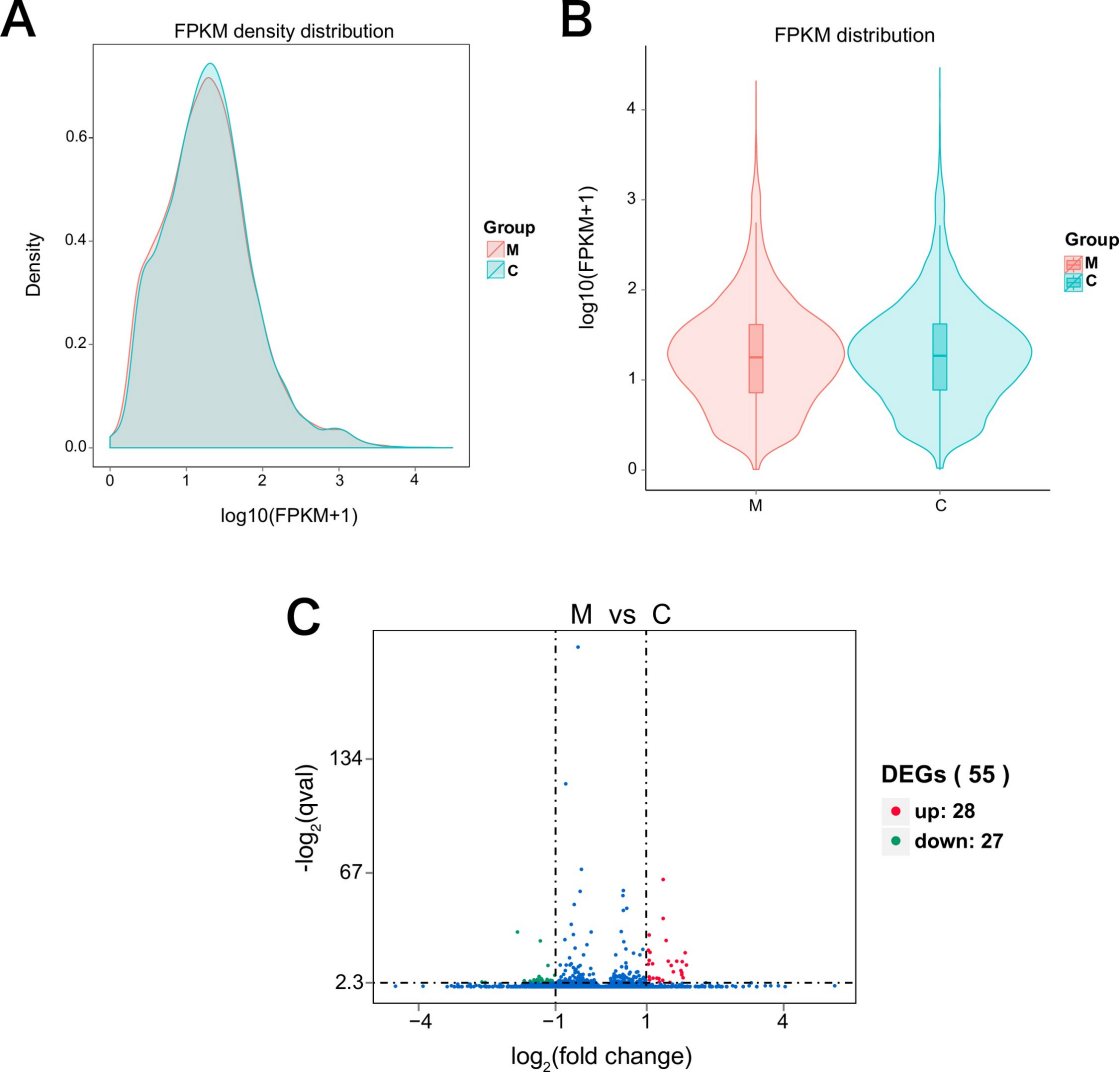
Comparisons of the FPKM values between mannitol-fed and carbohydrate-free conditions. M, *T*. *castaneum* adults fed gypsum supplemented with 200 mM mannitol for 48 h; C, *T*. *castaneum* adults fed gypsum without any carbohydrates for 48 h. (A) FPKM density distribution. The x-axis shows the log10(FPKM+1) and the y-axis shows gene density. (B) FPKM violin Plot. The x-axis shows the sample names and the y-axis shows the log10(FPKM+1). Each violin has five statistical magnitudes (maximum value, upper quartile, median, lower quartile and minimum value). The violin width shows the gene density. (C) The x-axis shows the fold change in gene expression between different samples, and the y-axis displays the statistical significance of the differences. Significantly up and down regulated genes are highlighted in red and green, respectively. Genes that did not express differently between treatment group and control group are shown in blue.

For DEGSeq without biological replicates, prior to differential gene expression analysis, for each sequenced library, the read counts were adjusted by the edgeR program package with one scaling normalization factor. The DEGSeq software was used to perform differential gene expression analysis between carbohydrate-free and mannitol-fed *T*. *castaneum*; the threshold was set to the usual padj < 0.05 [28]. The mRNA mean reads were normalized to FPKM values. Comparisons of the FPKM values between M (mannitol-fed) and C (carbohydrate-free) showed a relatively higher mRNA expression level in the mannitol-fed conditions. The density distribution (Fig 5A) and the violin diagram (Fig 5B) of FPKM visually demonstrate the gene expression levels. Volcano plots were used to infer the overall distribution of differentially expressed genes (Fig 5C). The threshold was normally set as: |log2(Fold Change)| > 1 and *q*-value < 0.005. Twenty-eight genes were upregulated for M compared to C, whereas 27 genes were downregulated (Fig 5C). These results indicate that even after being fed mannitol, most genes in *T*. *castaneum* have little effect, when compared with their effects under carbohydrate-free conditions. Differentially expressed upregulated genes are shown in Table 1 and downregulated genes in S4 Table, with gene ontology (GO) annotation. Additionally, KEGG pathway analysis of the differentially expressed genes provided significant insight into the potential biological pathways activated in response to mannitol feeding. The upregulated genes belong to various functional categories such as fatty acid synthesis, pyruvate metabolism, stress proteins and carbohydrate metabolism (S1–S5 Figs). Feeding on mannitol allowed *T*. *castaneum* adults to extend their lifespans compared to that of adults fed carbohydrate-free gypsum (Fig 1B). The results suggested that the extended lifespan was provided by upregulated genes related to the metabolism of energy-providing food sources such as fatty acids and carbohydrates.

**Table 1:** Upregulated genes.

Gene ID	log2.Fold_change.	p value	GO term
663216	1.8634	9.09E-16	alpha-crystallin A chain
660272	1.8311	3.01E-23	trans-1%2C2-dihydrobenzene-1%2C2-diol dehydrogenase
657759	1.7849	4.24E-08	thymidylate synthase
663240	1.7699	5.92E-18	protein lethal(2)essential for life
660669	1.7566	7.87E-10	cathepsin L precursor
100142317	1.7551	3.00E-11	heat shock protein 68a
660153	1.7446	2.18E-12	protein lethal(2)essential for life
100142517	1.651	3.46E-18	heat shock protein 68b
103312428	1.5694	8.79E-12	leucine-rich repeat-containing protein 15
662237	1.5234	1.46E-15	protein lethal(2)essential for life
663954	1.5018	8.38E-05	alpha amylase
657939	1.4611	3.46E-18	Z9 acyl-CoA desaturase B
658103	1.4169	1.12E-30	Z9 acyl-CoA desaturase A
103314884	1.3526	2.58E-67	fatty acid synthase
663483	1.3516	8.87E-44	pathogenesis-related protein 5
663852	1.3382	1.66E-06	mitochondrial sodium/hydrogen exchanger 9B2%2C transcript variant X4
661282	1.2667	3.95E-05	cytochrome P450 9AB1
660916	1.2532	2.07E-07	proline-rich extensin-like protein EPR1
662620	1.2095	9.48E-08	hexamerin 5
659356	1.1292	1.27E-07	alpha-trehalose-phosphate synthase
100142345	1.1227	1.00E-16	putative uncharacterized protein ART2
661091	1.0655	1.33E-23	sorbitol dehydrogenase
661777	1.0511	1.52E-06	uncharacterized LOC661777%2C transcript variant X1
103314314	1.0462	1.06E-18	brachyurin
655734	1.0423	5.33E-34	transferrin
660776	1.0402	1.24E-08	putative tricarboxylate transport protein%2C mitochondrial
656728	1.0237	1.87E-16	ATP-citrate synthase%2C transcript variant X1
658362	1.021	6.51E-25	acetyl-CoA carboxylase%2C transcript variant X6

### Verification of transcript expression levels using quantitative RT-PCR

**Fig 6:**
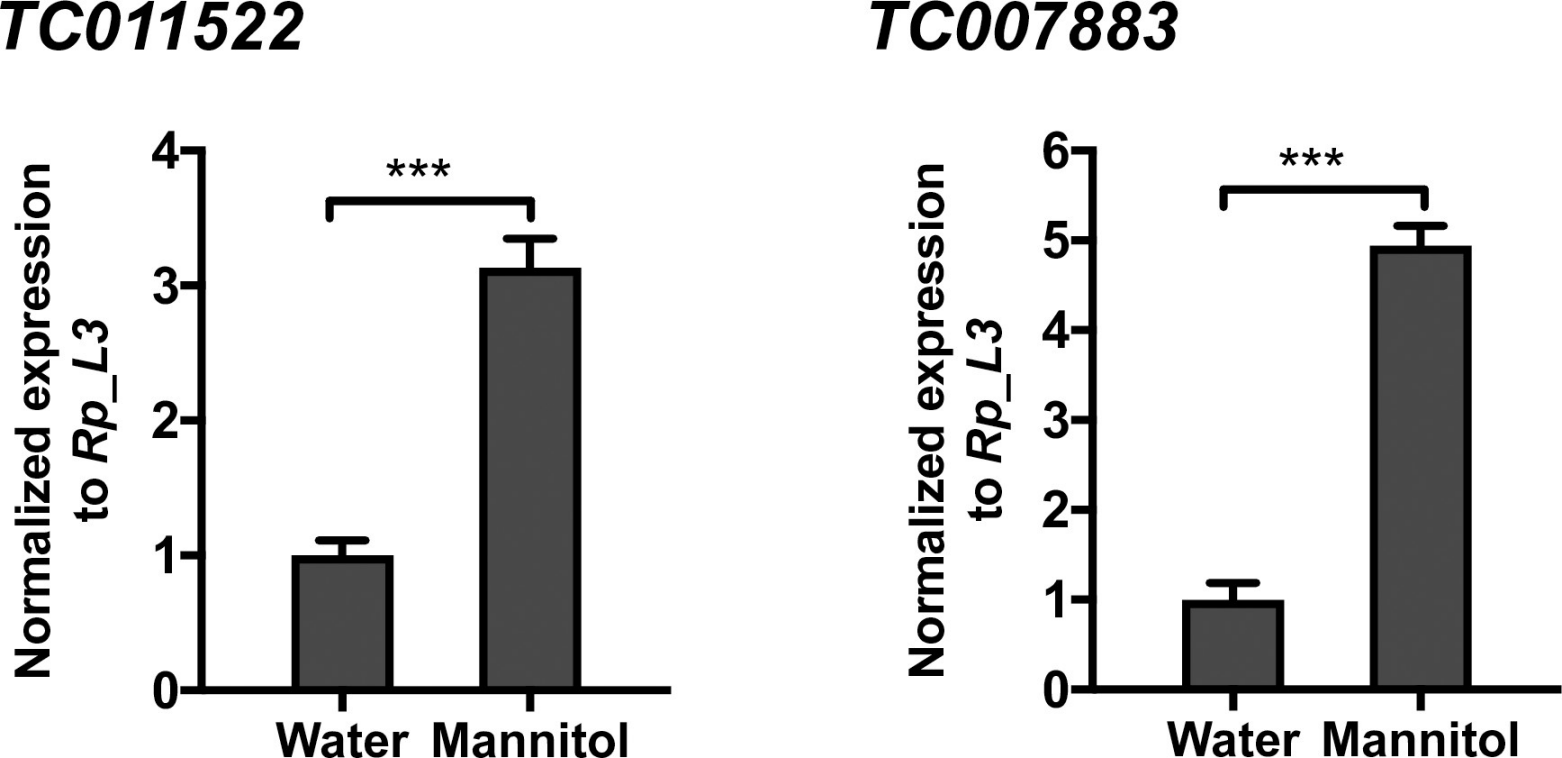
Validation of mannitol-responsive genes by quantitative RT-PCR. The relative expression levels of candidate genes for mannitol metabolism were determined by quantitative RT-PCR. TC011522, fatty acid synthase; TC007883, alpha-trehalose-phosphate synthase. Relative expression was calculated using the 2-*ΔΔ*Ct method. Ribosomal protein S3 (*RpS3*) in *T*. *castaneum* was used as the control to normalize the amount of templates. Error bars show S.E.M (n = 3). The statistical significance was determined using the *t*-test (***, *P* = 0.001 for TC011522; *P* = 0.0002 for TC007883).

To validate the mRNA expression profiles using RNA-Seq, mannitol-responsive genes associated with fatty acid synthesis and trehalose synthesis were analyzed by quantitative RT-PCR (Fig 6). The gene IDs were TC011522, encoding fatty acid synthase, and TC007883, encoding alpha-trehalose-phosphate synthase, respectively. Similar expression patterns of both genes were also obtained using quantitative RT-PCR, validating the reproducibility of gene expression data in this study.

## Discussion

This study shows that mannitol, a facilitator of feeding behavior of *T*. *castaneum* adults, acts as a nutrient source.

Thus far, it remained unclear whether *T*. *castaneum* uses mannitol as a nutrient. The lifespan of *T*. *castaneum* fed a diet of gypsum supplemented with mannitol as the sole carbon source was extended when compared with the lifespan of beetles fed on carbohydrate-free gypsum diet (Fig 1B). Mannitol probably can provide effective nutrition for *T*. *castaneum* adults. The mannitol content in the diet was clearly decreased after gypsum was excreted as a waste product (Fig 2). The mannitol derived from the diet would be transported to the hemolymph via epithelial gut cells expressing transporters. Aquaporin is a water molecule transporter, which is also responsible for the transport of eight polyols including mannitol across the transmembrane [29]. Several aquaporin genes were expressed in the gut in *T*. *castaneum* [30], possibly allowing the aquaporin to transport mannitol from the gut cavity to the hemolymph. After being fed mannitol the hemolymph of *T*. *castaneum* adults did not contain mannitol. Rather, trehalose was detected in the hemolymph despite the presence of mannitol in the diet (Fig 3A), indicating that mannitol was possibly metabolized to trehalose. When *T*. *castaneum* adults were fed gypsum in the absence of carbohydrates (i.e., water only), no sugars could be detected in the hemolymph except for a small amount of glucose. These results suggest that almost all hemolymph sugars were consumed as a carbon source during the food-deprived periods, being indispensable for activity prior to the dietary intake assay using gypsum (Fig 3B). The diet containing mannitol led to an increase of trehalose in the hemolymph of *T*. *castaneum* adults (Fig 3A).

Trehalose, a non-reducing disaccharide composed of two glucose molecules with an α-1,1-glycosidic bond, is synthesized in the fat body [31, 32]. Trehalose is exported via the facilitated trehalose transporter TRET1 to the hemolymph [33], and thus is a major hemolymph sugar in many insects [31]. Trehalose synthesis in insects is promoted by two key enzymes, trehalose-6-phosphate synthase (TPS, EC 2.4.1.15) and trehalose-6-phosphate phosphatase (TPP, EC 3.1.3.12). TPS catalyzes from UDP-glucose and glucose-6-phosphate (G6P) to trehalose-6-phosphate (T6P). Next, TPP catalyzes the dephosphorylation of T6P to trehalose [32]. A *TPP* gene is lacking in the genome database of *T*. *castaneum*, indicating that this beetle only has one *TPS* gene, which can synthesize trehalose only by acting as a bifunctional enzyme governing both TPS and TPP [18, 34]. The *TPS* gene was upregulated by mannitol in the diet (Table 1 and Fig 6), showing that it will facilitate the transition from mannitol to trehalose, while glycogen, a polysaccharide accumulating as carbohydrate storage in the fat body, was undetectable in a colorimetric assay (S1 File). The genes involving glycogen synthesis were not upregulated by mannitol in the diet. Glycogen is degraded by some enzymes such as glycogen phosphorylase, glucophosphatase and UDP-glucose pyrophosphorylase to UDP-glucose and G6P, resulting in the enhanced production of trehalose [35, 36]. The mannitol intake by *T*. *castaneum* adults increased *TPS* expression, and thereby may not accelerate the pathway to glycogen synthesis.

Some dietary carbohydrates were not only transformed to trehalose and glycogen, but also to lipids as the main component in the fat body. In insects, over 90% of the stored lipids consists of triglycerides [37, 38]. Fatty acids are rapidly taken up by the fat body and readily incorporated into triglycerides, which are utilized for energy production through *β*-oxidation [38, 39]. The rate of fatty acid synthesis was greatly accelerated when a sugar diet was provided, but not by the addition of starch [40]. In this study, the fatty acid synthesis pathway was also found to be activated by a mannitol-containing diet relative to a carbohydrate-free one (Table 1). In addition, triglyceride in the fat body was markedly increased by mannitol in the diet (Fig 4B), indicating that mannitol taken up in the body would be transferred to storage as triglyceride. The triglyceride content was maintained when the beetles were fed wheat-flour, whereas, like trehalose in the hemolymph, it was markedly decreased under food-deprived conditions (Fig 4A). These results suggest that triglyceride stored as an energy reserve in the fat body was mostly consumed during periods of starvation to supply material for the manufacture of products such as ATP, and began to be restored upon mannitol intake. In *Drosophila*, some genes implicated in fatty acid synthesis, including acetyl CoA carboxylase and ATP citrate lyase, were upregulated when the diet contained sugar, but remained unchanged under starvation [17]. Similar results were obtained when mannitol was provided (Table 1 and S2 and S3 Figs). The mannitol-responsive metabolism of *T*. *castaneum* is comparable to the expression of sugar-responsive genes in *Drosophila*.

In several insects, a positive effect of mannitol as a nutritive agent has not been documented. Mannitol reduced the lifespan of *Pimpla turionellae* adults, when compared with sucrose as a representative nutritive sugar, and did not permit the reproduction of adult females [41]. Mannitol also acts as a poisonous agent for *Drosophila* females because it reduces their lifespan markedly rather than being nutritious [42]. If this negative physiological effect of mannitol is be observed in *T*. *castaneum* adult females, it could be used as a bait pesticide for this beetle. However, mannitol is not poisonous but nutritive for *T*. *castaneum*. Therefore, it will be difficult to utilize mannitol as a bait insecticide in pest control applications.

Although this study, by analyzing mannitol-responsive gene expression profiles using RNA-Seq, revealed that mannitol is indeed utilized indeed by *T*. *castaneum*, it raised another question, i.e., how is mannitol metabolized to other carbohydrates. Actually, the enzymes related to the catalysis of mannitol to fructose and/or mannitol-1-phosphate are still unknown in the KEGG database (S1 Fig). Given that trehalose and triglyceride increased upon mannitol intake, mannitol must be metabolized to other carbohydrates. One possible pathway is the one involving sorbitol dehydrogenase (SDH, EC 1.1.1.14), an enzyme converting the sorbitol of polyols to fructose, which may also function in mannitol metabolism. SDH catalyzes mannitol, an isomer of sorbitol, at an even lower activity than it does sorbitol [43, 44]. As another possibility, mannitol catalysis is promoted by NADP^+^-dependent D-arabitol dehydrogenase. Arabitol dehydrogenase was identified in the rust fungus *Uromyces fabae* [45]. Highly homologous genes (Gene ID: LOC656777, LOC656868 and LOC656956) were retrieved from the *T*. *castaneum* genome using BLAST analysis, indicating that these genes may encode mannitol metabolism like in the rust fungus. Further studies of mannitol metabolism in *T*. *castaneum* are required to test these hypotheses.

## Supporting information

S1 Table Primers for quantitative RT-PCR. (PDF)

S2 Table Data quality. (PDF)

S3 Table Mapping status to *T*. *castaneum* reference genome. (PDF)

S4 Table Down-regulated genes. (PDF)

S1 Fig Fructose and mannose metabolism. (PDF)

S2 Fig Pyruvate metabolism. (PDF)

S3 Fig Fatty acid biosynthesis. (PDF)

S4 Fig Biosynthesis of unsaturated fatty acids. (PDF)

S5 Fig Starch and sucrose metabolism. (PDF)

S6 Fig Protein processing in endoplasmic reticulum. (PDF)

S1 File Raw data. (XLSX)

## Abbreviations

ORF: open reading frame
RT: reverse transcription
TribUTE: *Tribolium* Urges To Eat
DRA: DDBJ sequence read archive
TPS: Trehalose-6-phosphate synthase
KEGG: Kyoto Encyclopedia of Genes and Genomes
GO: gene ontology
DEG: differentially expressed genes
FPKM: Fragments Per Kilobase of transcript sequence per Millions base pairs sequenced
G6P: glucose-6-phosphate
