# Supplementary material for: Response of Tribolium castaneum to dietary mannitol, with remarks on its possible nutritive effects
Source: PLoS One. 2018 Nov 14;13(11):e0207497. doi: 10.1371/journal.pone.0207497 (PMC6235386; doi:10.1371/journal.pone.0207497)
Supplement: S1 Table — (PDF) [file pone.0207497.s001.pdf]

S1 Table Primers for quantitative RT-PCR

Name	Direction	Sequence
RpS3_f1	forward	TGGCGATGGCGTTTTCAAAG
RpS3_r1	reverse	ATGATTTCCGTGCGAGTTGG
TPS_f1	forward	AGATGTGATCCGCTTTTGAC
TPS_r1	reverse	CCGGAGGAGGCGACGAC
FAS_f1	forward	AAAACCGGGCAATGTGATGCC
FAS_r1	reverse	AAATGCCTTGCACATGCCTTG
