# Supplementary material for: Response of Tribolium castaneum to dietary mannitol, with remarks on its possible nutritive effects
Source: PLoS One. 2018 Nov 14;13(11):e0207497. doi: 10.1371/journal.pone.0207497 (PMC6235386; doi:10.1371/journal.pone.0207497)
Supplement: S2 Table — (PDF) [file pone.0207497.s002.pdf]

S2 Table Data quality

Sample	Raw reads	Clean reads	Clean base (G)	Error rate (%)	Q20 (%)	Q30 (%)	GC content (%)
carbohydrate-free	25,203,990	24,744,966	3.7	0.03	94.64	87.47	44.21
mannitol	25,708,420	24,303,030	3.6	0.02	95.32	89.2	43.07

Sample name: the names of samples
Raw Reads: the original sequencing reads counts
Clean Reads: number of reads after filtering
Clean Bases: clean reads number multiply read length, saved in G unit
Error Rate: average sequencing error rate, which is calculated by Qphred=-10log10(e)
Q20: percentages of bases whose correct base recognition rates are greater than 99% in total bases
Q30: percentages of bases whose correct base recognition rates are greater than 99.9% in total bases
GC content: (G & C base count) / (Total base count)
