# Supplementary material for: Response of Tribolium castaneum to dietary mannitol, with remarks on its possible nutritive effects
Source: PLoS One. 2018 Nov 14;13(11):e0207497. doi: 10.1371/journal.pone.0207497 (PMC6235386; doi:10.1371/journal.pone.0207497)
Supplement: S3 Table — (PDF) [file pone.0207497.s003.pdf]

S3 Table Mapping status to *T. castaneum* reference genome

**Sample_name**	**M**	**C**
Total reads	24744966	24303030
Total mapped	14126678 (57.09%)	13566706 (55.82%)
Multiple mapped	92002 (0.37%)	92658 (0.38%)
Uniquely mapped	14034676 (56.72%)	13474048 (55.44%)
Reads map to '+'	7248180 (29.29%)	7024634 (28.9%)
Reads map to '-'	6786496 (27.43%)	6449414 (26.54%)
Non-splice reads	9076369 (36.68%)	8748516 (36%)
Splice reads	4958307 (20.04%)	4725532 (19.44%)
Mapped to Exon (%)	97.3	96.8
Mapped to Intron (%)	1.6	1.9
Mapped to Intergenic (%)	1.1	1.4

Total number of filtered reads (Clean data).
Total number of reads that can be mapped to the reference genome.
Number of reads that can be mapped to multiple sites in the reference genome. This number is usually less t
Number of reads that can be uniquely mapped to the reference genome.
Number of reads that map to the positive strand (+) or the minus strand (-).
Splice reads can be segmented and mapped to two exons (also named junction reads), whereas non-splice
The ratio of splice reads depends on the insert size used in the RNA-seq experiments.
