# Supplementary material for: Response of Tribolium castaneum to dietary mannitol, with remarks on its possible nutritive effects
Source: PLoS One. 2018 Nov 14;13(11):e0207497. doi: 10.1371/journal.pone.0207497 (PMC6235386; doi:10.1371/journal.pone.0207497)
Supplement: S4 Table — (PDF) [file pone.0207497.s004.pdf]

S4 Table Down-regulated genes

Gene ID	log2.Fold_change.	p value	GO term
100142233	-1.5614	8.8911E-06	dynein assembly factor 1%2C axonemal
103312779	-1.3065	1.4037E-06	uncharacterized LOC103312779
103312783	-1.4854	9.3439E-07	CRISP/Allergen/PR-1
103312954	-1.2364	0.000052209	early endosome antigen 1-like%2C transcript variant X3
103313005	-1.0281	1.6323E-09	uncharacterized LOC103313005
103313194	-1.4364	0.000018617	uncharacterized LOC103313194
103313290	-1.3252	1.8974E-07	uncharacterized LOC103313290
103314745	-1.1907	0.00003287	uncharacterized LOC103314745
103314997	-1.541	0.000059798	uncharacterized LOC103314997
103315014	-1.4049	5.4528E-07	hypothetical protein%2C transcript variant X2
103315104	-1.1911	6.0879E-07	hypothetical protein
107398123	-1.1745	1.4987E-15	neurofilament heavy polypeptide-like
657573	-1.3419	1.7615E-30	uncharacterized LOC657573%2C transcript variant X1
658313	-1.1176	2.4805E-06	sperm flagellar protein 1
659448	-1.6925	3.3907E-06	coiled-coil domain-containing protein 96
659597	-1.3689	8.5381E-09	dynein heavy chain 10%2C axonemal
660270	-1.8464	1.2534E-35	cytochrome P450-like protein
660662	-1.1314	0.000007237	protein I'm not dead yet
661075	-1.2251	0.00002887	kelch-like protein 10%2C transcript variant X3
661455	-2.5493	0.000084219	venom acid phosphatase Acph-1
661496	-2.6235	0.000023578	venom acid phosphatase Acph-1
661925	-1.2861	0.000055713	protein Cep78 homolog%2C transcript variant X2
662770	-1.3785	4.1485E-06	facilitated glucose transporter protein 1
662967	-1.1905	0.000022102	IQ and AAA domain-containing protein 1-like
663124	-1.0854	4.8943E-06	EF-hand domain-containing family member C2
663637	-1.3005	4.4818E-06	dynein heavy chain 7%2C axonemal%2C transcript variant X1
663840	-1.4656	0.000045577	uncharacterized LOC663840
