# Supplementary figures and images for: Response of Tribolium castaneum to dietary mannitol, with remarks on its possible nutritive effects
Source: PLoS One. 2018 Nov 14;13(11):e0207497. doi: 10.1371/journal.pone.0207497 (PMC6235386; doi:10.1371/journal.pone.0207497)

## FRUCTOSE AND MANNOSE METABOLISM

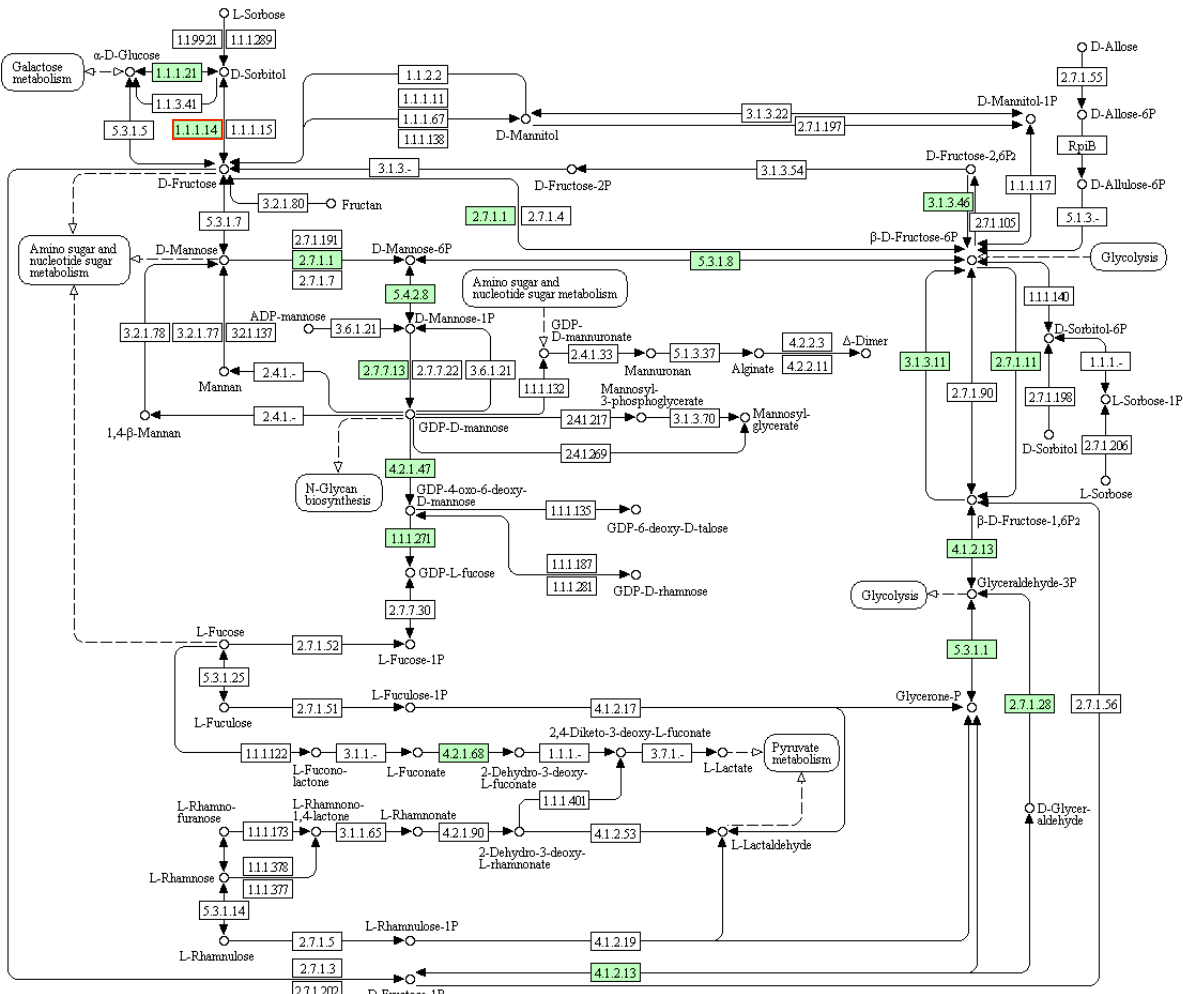

Supplement: S1 Fig — (PDF) [file pone.0207497.s005.pdf]

# PYRUVATE METABOLISM

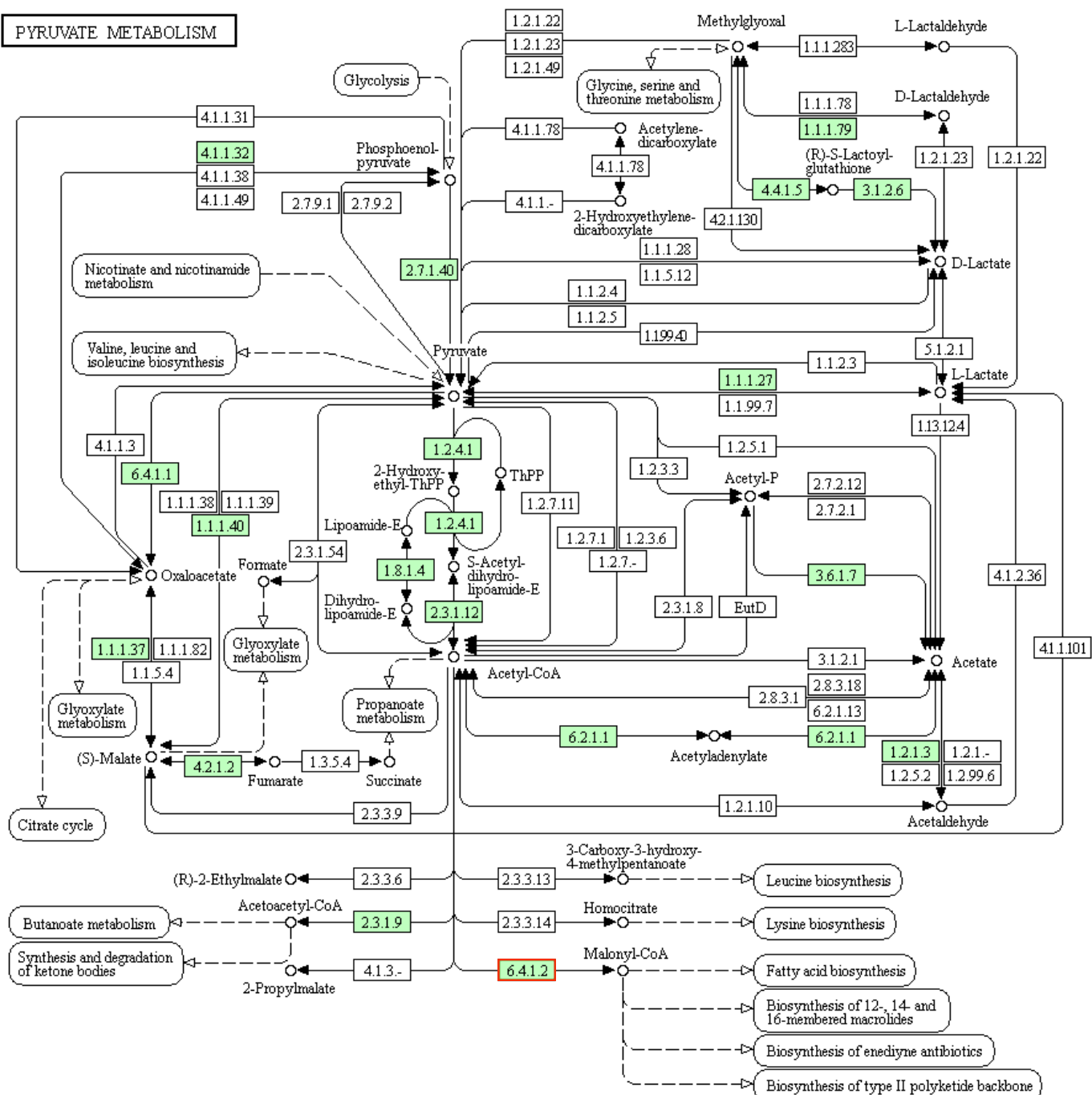

Supplement: S2 Fig — (PDF) [file pone.0207497.s006.pdf]

# FATTY ACID BIOSYNTHESIS

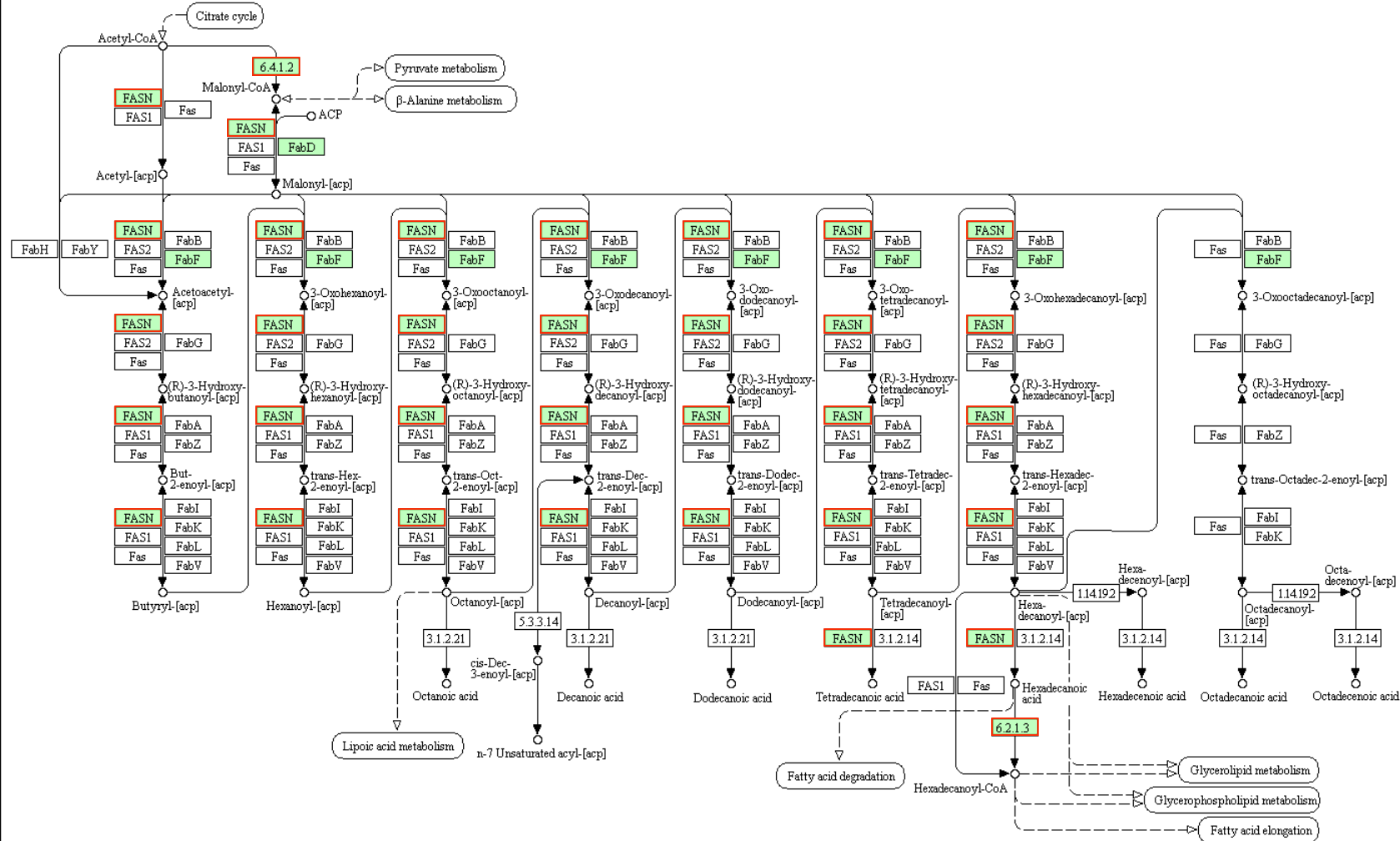

Supplement: S3 Fig — (PDF) [file pone.0207497.s007.pdf]

# BIOSYNTHESIS OF UNSATURATED FATTY ACIDS

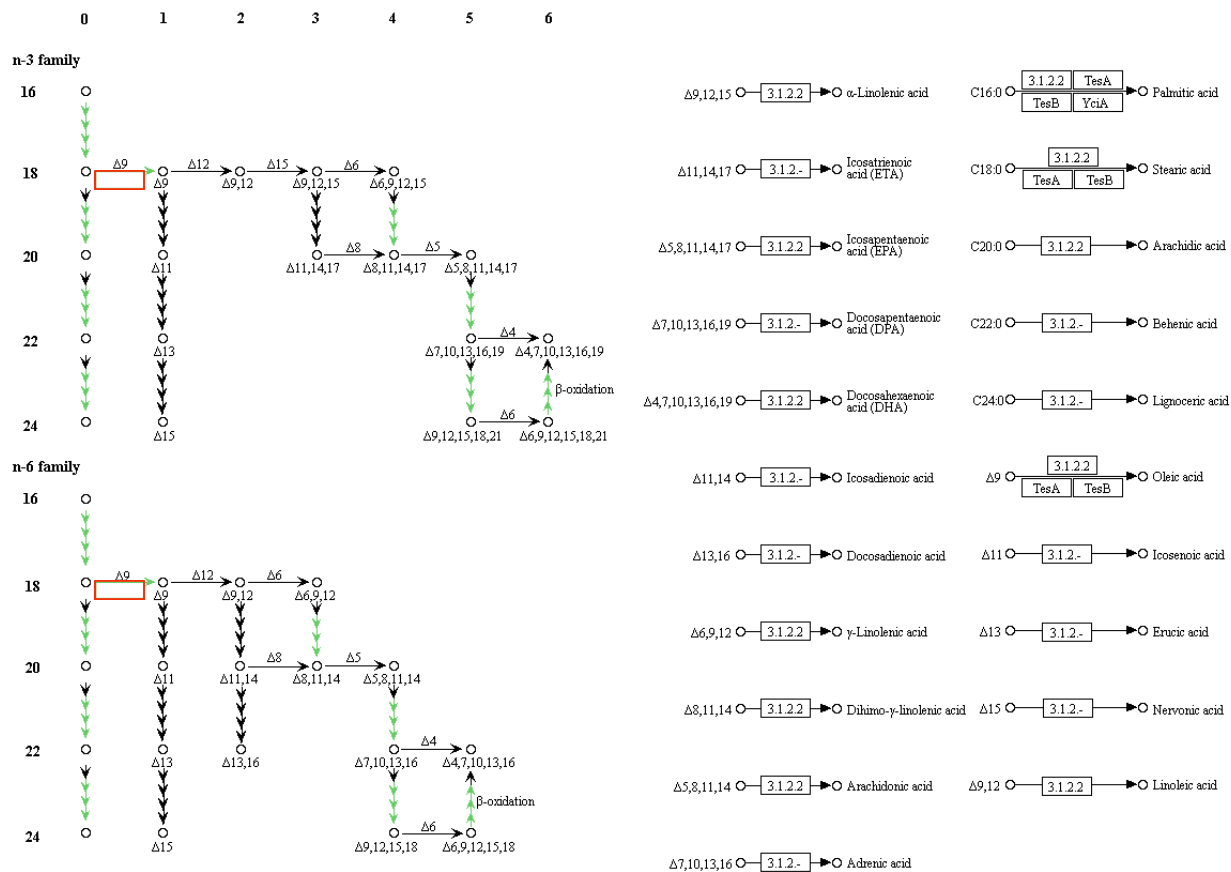

Supplement: S4 Fig — (PDF) [file pone.0207497.s008.pdf]

## STARCH AND SUCROSE METABOLISM

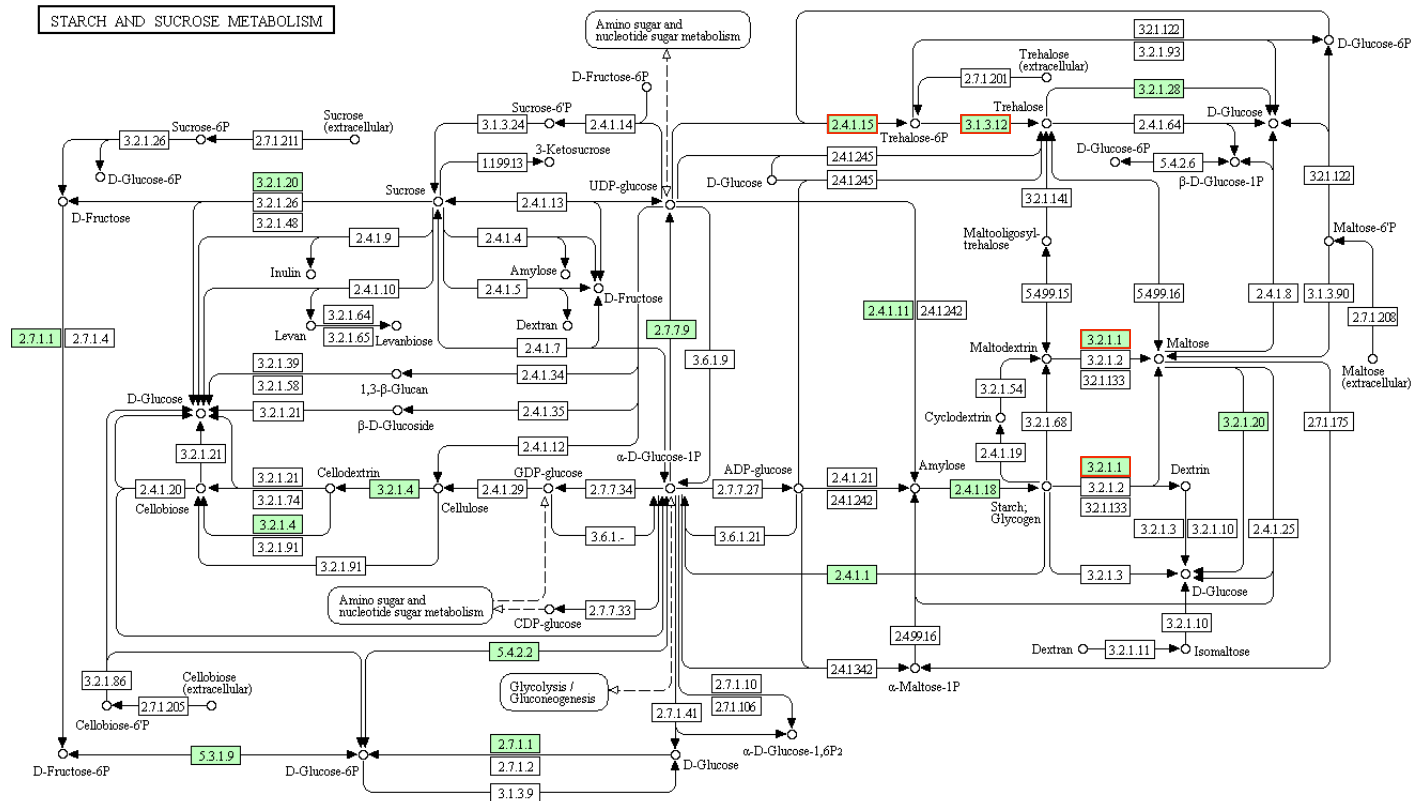

Supplement: S5 Fig — (PDF) [file pone.0207497.s009.pdf]

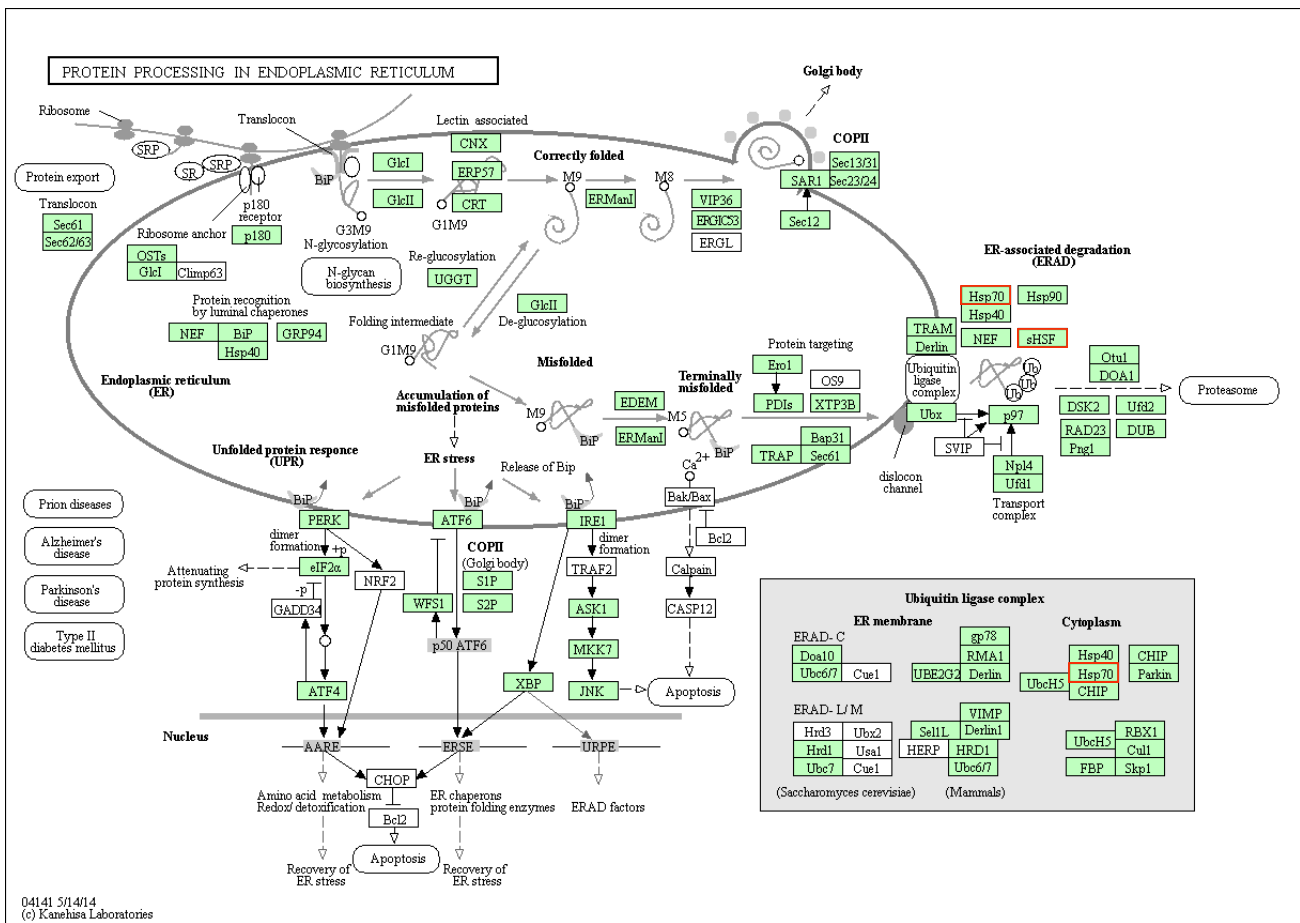

Supplement: S6 Fig — (PDF) [file pone.0207497.s010.pdf]
